# The effects of curcumin (diferuloylmethane) on body composition of patients with advanced pancreatic cancer

**DOI:** 10.18632/oncotarget.7773

**Published:** 2016-02-27

**Authors:** Henrique A. Parsons, Vickie E. Baracos, David S. Hong, James Abbruzzese, Eduardo Bruera, Razelle Kurzrock

**Affiliations:** ^1^ Department of Medicine/Division of Palliative Care, University of Ottawa, Ontario, Canada; ^2^ Department of Oncology/Division of Palliative Care Medicine, University of Alberta, Edmonton, Alberta, Canada; ^3^ Department of Investigational Cancer Therapeutics (A Phase I Clinical Trials Program), The University of Texas M. D. Anderson Cancer Center, Houston, Texas, USA; ^4^ Department of Medicine/Division of Oncology Duke University School of Medicine, Durham, North Carolina, USA; ^5^ Department of Palliative Care and Rehabilitation Medicine, The University of Texas M. D. Anderson Cancer Center, Houston, Texas, USA; ^6^ Division of Hematology & Oncology and Center for Personalized Cancer Therapy University of California, San Diego, Moores Cancer Center, San Diego, California, USA

**Keywords:** pancreatic neoplasms, curcumin, body composition, inflammation

## Abstract

**Background:**

Curcumin is a natural product that is often explored by patients with cancer. Weight loss due to fat and muscle depletion is a hallmark of pancreatic cancer and is associated with worse outcomes. Studies of curcumin's effects on muscularity show conflicting results in animal models.

**Methods and results:**

Retrospective matched 1:2 case-control study to evaluate the effects of curcumin on body composition (determined by computerized tomography) of 66 patients with advanced pancreatic cancer (22 treated,44 controls). Average age (SEM) was 63(1.8) years, 30/66(45%) women, median number of prior therapies was 2, median (IQR) time from advanced pancreatic cancer diagnosis to baseline image was 7(2-13.5) months (p>0.2, all variables). All patients lost weight (3.3% and 1.3%, treated vs. control, p=0.13). Treated patients lost more muscle (median [IQR] percent change −4.8[−9.1,-0.1] vs. −0.05%[−4.2, 2.6] in controls,p<0.001) and fat (median [IQR] percent change −6.8%[−15,-0.6] vs. −4.0%[−7.6, 1.3] in controls,p=0.04). Subcutaneous fat was more affected in the treated patients. Sarcopenic patients treated with curcumin(n=15) had survival of 169(115-223) days vs. 299(229-369) sarcopenic controls(p=0.024). No survival difference was found amongst non-sarcopenic patients.

**Conclusions:**

Patients with advanced pancreatic cancer treated with curcumin showed significantly greater loss of subcutaneous fat and muscle than matched untreated controls.

## INTRODUCTION

Cancer cachexia is “a multi-factorial syndrome defined by an ongoing loss of skeletal muscle mass (with or without loss of fat mass) that cannot be fully reversed by conventional nutritional support and leads to progressive functional impairment. The pathophysiology is characterized by a negative protein and energy balance driven by a variable combination of reduced food intake and abnormal metabolism” [1, 2]. The majority of patients with cancer lose weight at the end of life [3, 4], and approximately 20% of all cancer deaths are related to cachexia [5].

Loss of body mass during the cancer trajectory has been associated with worse outcomes such as decreased survival in patients with pancreatic cancer, loss of physical strength, and poorer response to therapy [3, 6–9]. In the setting of pancreatic cancer, a 10% weight loss in comparison with premorbid weight is present in around 80% of all cases, and at least 25% of these cases meet the definition of cancer cachexia [7, 10].

There is a dearth of effective interventions to treat cancer cachexia, and several approaches are currently being researched. Since inflammation is thought to have a pivotal role in the genesis of cancer cachexia [11], agents that target inflammatory pathways are of special interest. Curcumin (diferuloylmethane), the phytochemical component responsible for the characteristic yellow-gold color of turmeric (a spice used mostly in Asia) is found in the root of the *Curcuma longa* plant [12] and has a myriad of biologic properties [13–15], including antineoplastic [16–19] and anti-inflammatory capabilities [20–22]. It is a popular natural product that is of interest to many patients with cancer.

Given the clear necessity for new therapeutic options for cancer cachexia, especially in patients with pancreatic cancer, and considering the anti-inflammatory actions of curcuminoids [20–22], we conducted a study to evaluate the effects of curcumin on body composition of such patients. Specifically, we aimed to (1) determine how body composition (namely body fat and muscle) evolve over time in patients with advanced pancreatic cancer treated with curcumin, and (2) determine whether there are different body composition changes over time in patients with advanced pancreatic cancer who received curcumin compared with matched patients who did not receive this agent.

## RESULTS

**Figure 1:**
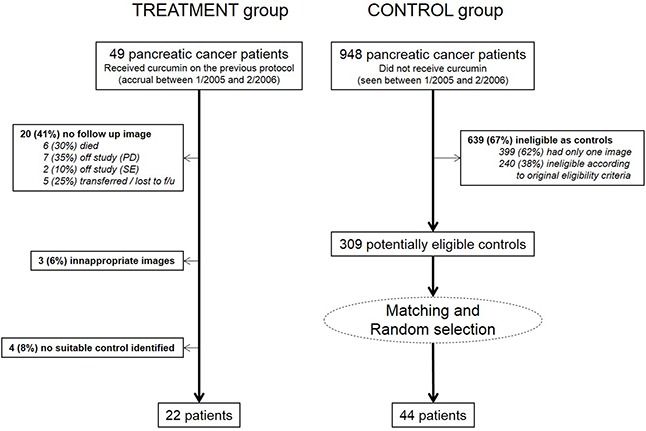
Study group accrual information (PD=Progressive disease; SE=stable disease)

A total of 66 patients were included in the current study. The treatment group was composed of 22 patients who received curcumin on a previous clinical. The control group was obtained from a pool of 948 patients with pancreatic cancer seen at our hospital in the same time period that the original clinical trial was accruing patients. Of those, 639 (67%) did not meet eligibility criteria either for the current or for the original protocol and were excluded. The final control group was composed of 44 patients randomly selected from the pool of 309 potentially eligible patients, matched with the patients in the treatment group by age, gender, body mass index, time from advanced cancer diagnosis to baseline image, and number of prior therapies. Figure 1 summarizes the accrual process. The matched demographic characteristics of the study sample are shown in Table 1.

**Table 1:** Matching characteristics

	Treatment	Control	p value
	(N=22)	(N=44)	
Female Gender (n, %)	10 (45.5%)	20 (54.5%)	1.000
Age (years) (mean, SEM)	63.8 (2.2)	63.2 (1.3)	0.823
Body Mass Index (mean, SEM)	23.8 (0.6)	24.1 (0.4)	0.707
Number of prior therapies (median, IQR)	2 (1-3)	2 (1-2)	0.237
Time between advanced cancer and baseline image (months) (median, IQR)	7 (2-13.5)	6 (3-13.75)	0.749

*SEM, standard error of the mean; IQR, interquartile range*.

### Body composition

Ascites and/or peripheral edema were present in 4/22 (18%) patients in the treatment group and in 10/44 (23%) patients in the control group (p=0.759). Those patients were excluded from body mass analyses but were included in the body composition analyses. The majority of patients lost weight between baseline and follow up in both study groups, with a statistically insignificant greater frequency of weight loss in the treatment group [15/18 (83%) and 19/34 (56%) in the treatment and control groups, respectively, p=0.07]. Average baseline (standard error of the mean, SEM) weight was 69.4 (2.4) kg and 69.0 (2.2) kg for patients in the treatment and control groups at baseline, respectively (p=0.911), while at follow up it was 67.0 (2.2) kg and 67.9 (2.0) kg respectively (p=0.782). The absolute average weight loss in this timeframe was greater in the treatment group [2.4 kg (SEM 0.8)] in comparison with the control group [1.1 kg (SEM 0.6)], albeit not statistically significant (p=0.174). The average percent weight loss, while also greater in the treatment group (3.3% of the baseline weight versus 1.3% of the baseline weight for the control group), did not reach statistical significance (p=0.130). Weight change did not differ by gender.

Classifying patients with BMI ≥ 25 kg/m^2^ as overweight or obese, 6/18 (33%) treated patients and 12/34 (35%) controls were respectively classified as such at baseline (p=1.000) and 4/18 (22%) and 11/34 (32%) at follow up (p=0.532). 2/18 (11%) treated patients and 3/34 (9%) controls changed from overweight/obese to normal/underweight between baseline and follow up (p=1.000). Changes in the opposite direction did not occur. Changes in body mass index (BMI) evidently behave in the same way as those seen for weight. Patients in the treatment group had an absolute decrease of 0.8 (0.3) kg/m^2^ in BMI while the controls lost 0.4 (0.2) kg/m^2^ (p=0.160).

All body composition parameters decreased in both groups between baseline and follow up. Body composition data is summarized in Table 2. At baseline, no significant differences were found with regards to body composition variables between patients in the two study groups. At follow up, patients treated with curcumin showed a trend towards lower subcutaneous fat area at L3, total adipose area at L3 and total estimated body fat as compared to the patients in the control group (Table 2, p=0.054, 0.07, and 0.07, respectively).

**Table 2:** Body Composition Analysis

	Baseline			Follow up			p
	Treatment	Controls	p	Treatment	Controls		
	N=22	N=44		N=22	N=44		
	Median (IQR)	Median (IQR)		Median (IQR)	Median (IQR)		
**Skeletal muscle area at L3 (cm^2^)**	119.7 (104-145.7)	125.1 (108-146.7)	0.661	112.5** (95.6-137.5)	122.6 (102-149)	0.202
**Muscle index (cm^2^/m^2^)**	42.3 (37-46.7)	42.3 (37.6-47.4)	0.747	40.2** (35.6-45.4)	41.6 (37.5-46.8)	0.115
**Intramuscular adipose area at L3 (cm^2^)**	7.5 (3.4-12)	7.9 (5.4-11)	0.833	7.7 (2.5-10.5)	7.0 (4.9-13)	0.430
**Visceral adipose area at L3 (cm^2^)**	57.3 (36.4-133.2)	73.4 (44.7-116.6)	0.668	42.3* (26.2-101)	68.5* (35.7-104)	0.286
**Subcutaneous adipose area at L3 (cm^2^)**	95 (65.7-191.9)	120 (101.6-172.7)	0.331	72.7** (59.9-160.5)	116.7* (84.6-149.2)	0.054
**Total adipose area at L3 (cm^2^)**	208.9 (119-317.5)	228.4 (147.3-294.8)	0.732	137.8** (85.8-266.8)	217.8* (154.2-282.9)	0.07
**Estimated total lean body mass (kg)**	42 (37.3-49.8)	43.6 (38.5-50.1)	0.661	39.8** (34.7-47.3)	42.8 (36.7-50.8)	0.202
**Estimated total fat body mass (kg)**	20 (16.2-24.5)	20.8 (18.5-23.6)	0.732	17** (14.8-22.4)	20.4* (17.7-23.1)	0.07

statistically significant differences between baseline and follow up time points within study groups.

* p < 0.05;

** p<0.001

**Figure 2:**
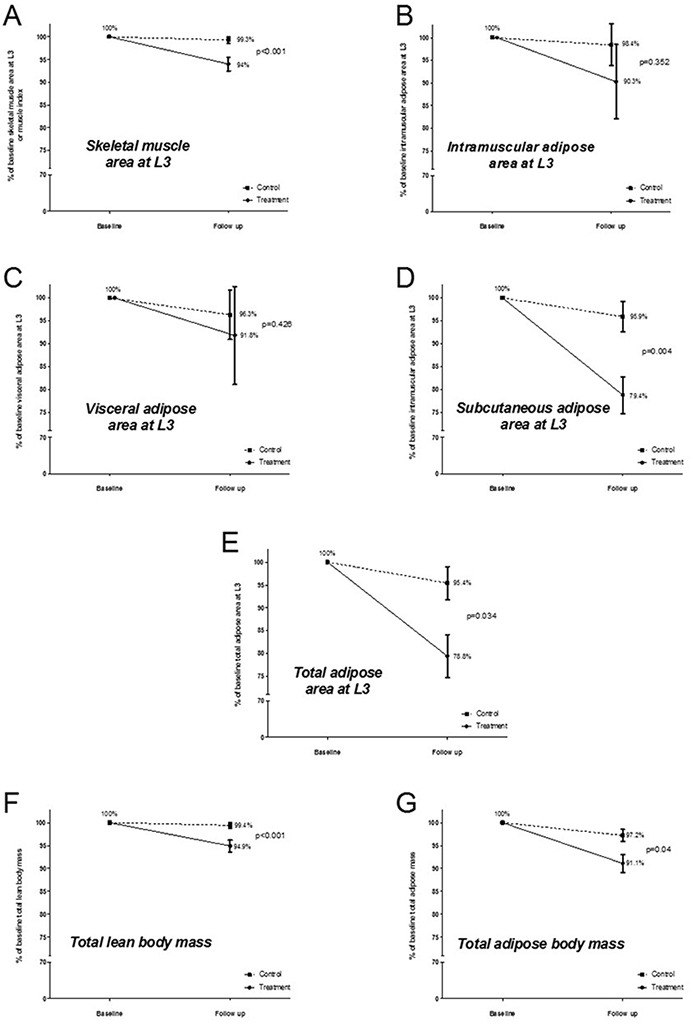
Average percent variation in body composition variables for the study groups (Whiskers represent the standard error of the mean)

Percent variation in body composition variables according to study groups is shown in Figure 2. Patients in the treatment group showed greater percent reduction in all parameters when compared to those in the control group. Significantly different reductions were observed for skeletal muscle area at L3, intramuscular adipose area at L3, total adipose area at L3, estimated total adipose body mass, and estimated total lean body mass. The median percent change in estimated total lean body mass and total adipose body mass was significantly greater for treated [−4.8% (IQR −9.1 to −0.1) and −6.8% (IQR −15 to −0.6), respectively] than for untreated patients [−0.05% (IQR −4.2 to 2.6) and −4.0% (IQR −7.6 to 1.3), respectively] (p<0.001 and p=0.04 for lean and fat body mass changes, respectively). The difference in percent changes for estimated total lean and adipose body masses was not statistically significant among curcumin treated patients, but was significantly different among the controls, with the fat loss being greater (p=0.03).

At baseline, sarcopenia was present in 15/22 (68%) treated patients and 27/44 (61%) controls (p=0.787) whereas at follow up, it was present in 18/22 (82%) treated and 29/44 (66%) untreated patients (p=0.252). The increase in sarcopenia frequency was not statistically significant in any of the groups (p=0.488 and p=0.825 for treated and untreated patients, respectively). No baseline sarcopenic patients reversed their low muscularity status at follow up. Sarcopenia co-occurred with overweight or obesity (BMI ≥ 25kg/m^2^) in 3/18 (17%) and 5/34 (15%) treated and untreated patients at baseline, respectively (p=0.574) and in 3/18 (17%) and 3/34 (9%) treated and untreated patients at follow up, respectively (p=0.339) (patients with ascites and/or peripheral edema were excluded). Male patients in the treatment group had a significantly greater frequency of sarcopenia as compared to female patients in the same group [11/12(92%) versus 4/10(40%) at baseline and 12/12 (100%) versus 6/10 (60%) at follow up (p=0.020 and 0.029, respectively)]. In the control group, the frequency of sarcopenia in males was also greater, but did not attain statistical significance at any of the time-points.

### Potential confounders

Several medications can impact body composition (especially affecting muscularity), and 4/22 (18%) of the treated patients used such drugs in the study period (progestin in three cases and testosterone in one), while 2/44 (4.5%) controls were receiving those medications (one progestin and one testosterone) (p=0.09). No patients were found to be under treatment with cannabinoids or corticosteroids in the study period. Among the treated patients, no difference was found between subjects who received other such drugs (ie progestins and corticosteroids) and those who did not receive them with regards to changes in average total lean body mass [−6.4% (SEM 3.5) vs. −4.8 (SEM 1.4), respectively, p=0.523] and total body fat [−9.9% (SEM 4.2) vs. −8.7 (SEM 2.3), respectively, p=0.58]. Statistical significance was not tested for the control patients due to the small number of subjects who received the drugs.

A proportion of patients in the control group received oncologic treatment in the study period (26/44, 59%). Gemcitabine, cisplatin, and oxaliplatin were the most common chemotherapeutics used. The percent change in total lean body mass was not statistically different between controls who received and did not receive oncologic treatment [−0.7% (SEM 0.8) vs. −0.5% (SEM 1.0), respectively, p=0.828]. Similarly, the percent change in total adipose body mass was not significantly different between controls according to oncologic treatment during the study period [−4.3% (SEM 1.5) vs. −0.7% (SEM 2.3), respectively, p=0.179].

### Survival analyses

**Figure 3:**
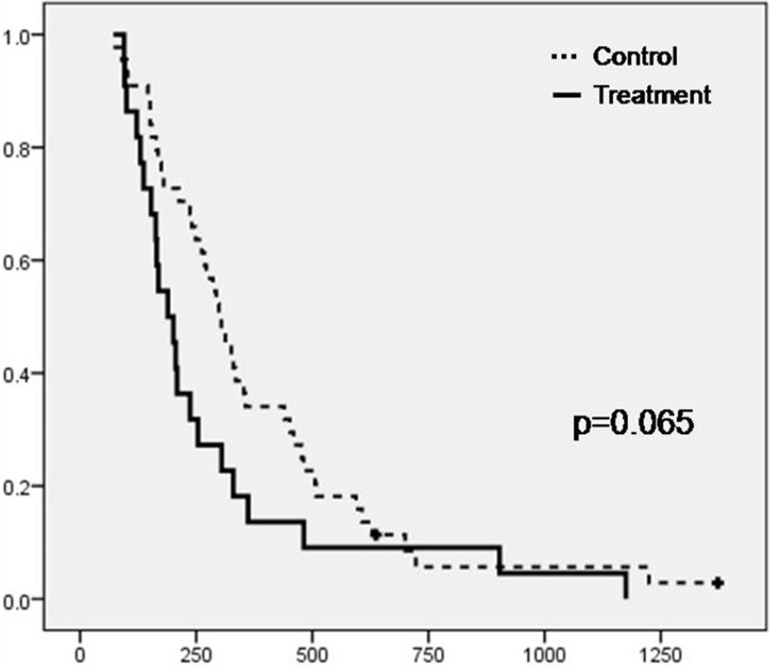
Kaplan-Meier plot depicting the survival from baseline of the patients in the two study groups (Treatment, N=22 and Control, N=44) (Crosses represent censored subjects)

Overall median survival from baseline (95% CI) was of 189 (142-236) days for the patients treated with curcumin and 299 (240-357) days for the patients in the control group (log rank p=0.065) (Figure 3). Survival was not significantly different between sarcopenic and non-sarcopenic patients overall [254 (216-291) vs. 293 (143-443) days, p=0.588]. However, when analyzed separately, the 15 sarcopenic patients in the treatment group showed significantly shorter survival [169 (115-223) days] in comparison with the 27 sarcopenic patients in the control group [299 (229-369) days, p=0.024], whereas no difference was found between the survival of the seven non-sarcopenic patients in the treatment group [254 (216-291)] and the 17 non-sarcopenic control patients [304 (184-423), p=0.910].

**Figure 4:**
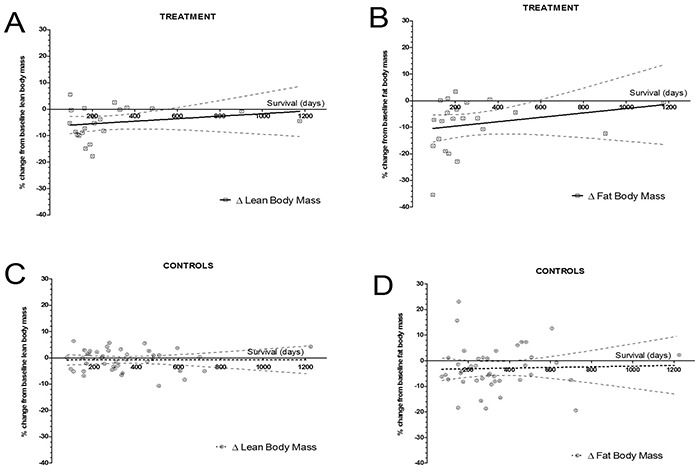
Percent change in adipose and lean body masses according to survival from baseline (Circles represent individual patients, solid line the regression line, and dashed lines the 95% confidence interval band). Spearman correlation coefficients between lean body mass change and survival were 0.283 and −0.035 (p=0.202 and 0.824) for patients treated with curcumin and controls, respectively (panels **A.** and **C.**). The Spearman correlation coefficients between fat body mass change and survival were 0.367 and 0.058 (p=0.09 and 0.713), for patients treated with curcumin and controls respectively (panels **B.** and **D.**).

Survival was plotted against changes in body composition between baseline and follow up for patients in the treatment and control groups whose death was confirmed (22/22 and 42/44, respectively) (Figure 4). The correlation between the variation of total lean body mass and survival yielded coefficients of 0.283 and −0.035 (p=0.202 and 0.824) for cases and controls, respectively, whereas the correlation between survival and variation in total fat body mass yielded coefficients of 0.367 and 0.058 (p=0.09 and 0.713) for cases and controls, respectively. Even though not statistically significant, shorter survival appeared to be correlated with greater reductions in body composition parameters only in the group of patients treated with curcumin.

## DISCUSSION

We have conducted a retrospective 1:2 matched “case control” study to evaluate the effects of curcumin on body composition of patients with advanced pancreatic cancer. We were not able to confirm our hypothesis that patients with advanced pancreatic cancer treated with curcumin for two months would have smaller losses in fat and muscle as compared to the matched controls not treated with curcumin. The sample of patients treated with curcumin for two months lost somewhat more weight than the controls, due to both fat and muscle losses.

Weight loss due to both fat and muscle depletion is common in patients with advanced pancreatic cancer. Wigmore et al. [23] showed, in a prospective observational study of 20 patients with advanced pancreatic cancer, that absolute fat and muscle losses measured by bioelectrical impedance are significantly different between diagnosis and death. Using the same retrospective CT analysis technique as this study, Tan et al. [24] described that the majority of patients with advanced pancreatic cancer lost body mass from both fat and muscle compartments as the disease evolved.

The different compartments of body composition, while consistently decreased, did so in a different fashion between groups in our study. Significant differences were found when comparing the percent reduction in areas of skeletal muscle and subcutaneous fat between treated and untreated patients. Therefore, both estimated total lean body mass and total adipose body mass showed significantly greater reductions in the treatment group. In addition, whereas patients in the control group lost significantly more adipose tissue than muscle, treated patients lost comparable percentages of muscle and fat.

Sarcopenia (decreased muscle mass) was present at baseline in 68% of the patients who received curcumin and 61% of the controls, increasing at follow up to 82% and 66% for treated and untreated patients, respectively. These figures are greater than the 51% and 47% prevalence that we have shown in samples of 104 and 306 patients with advanced cancer at our Phase I program, respectively [25, 26]. This is probably explained by the heterogeneous population in the previous studies. In the setting of pancreatic cancer, Joglekar et al. [27] studied 118 patients who underwent pancreatectomy and showed a prevalence of 26% of sarcopenic patients preoperatively; this population appeared to be at an earlier disease stage in comparison with our population. Tan et al. [24] reported that in a group of 44 pancreatic cancer patients, 46% were sarcopenic at the time of referral to a palliative care program and 61% were sarcopenic around 135 days later. Since our patients had a higher frequency of sarcopenia, it is plausible that their disease was more advanced. Interestingly, however, the median overall survival after the baseline image in Tan's study was the same as for the patients treated with curcumin in our study (189 days), and shorter than the median survival for patients in the control group (299 days). Therefore, it is likely that other factors are in play and affect the genesis of sarcopenia and/or survival in our patients. The frequency of sarcopenia increased in both groups at follow up, and no patients reversed the sarcopenic status. Therefore, it seems that curcumin did not attenuate sarcopenia in our limited sample.

Mounting evidence exists to support the potential effects of curcumin on the adipose tissue and more so in modulation of signal transduction pathways that are paramount for the generation of obesity and several of its complications [28, 29]. The present study showed that patients with advanced pancreatic cancer treated with curcumin had significantly greater losses of fat as compared to matched untreated controls, especially subcutaneous fat. This might indicate a direct effect of curcumin on adiposity which should be further explored in anti-obesity research.

Attenuation of weight loss by curcumin in the setting of pancreatic cancer was previously described in animal models only. One study in an animal model of cachexia (MAC16 colon tumor-bearing mice) showed that the administration of a 100mg/kg dose of a curcumin complex (curcumin c3, composed of 72% curcumin, 22% desmethoxycurcumin, and 4% bisdesmethoxycurcumin) was able to attenuate weight loss in the animals [30]. In addition to the fact that animal models frequently do not translate well to human clinical practice [31], it is of interest to note that the analogue composition of the drug used in the current study was somewhat different than the used in this animal study (87.2% curcumin, 10% desmethoxycurcumin, and 2.3% bisdesmethoxycurcumin in our study and 72% curcumin, 22% desmethoxycurcumin, and 4% bisdesmethoxycurcumin in the animal study) [32], which might also contribute for the different findings. Indeed, it appears that each curcumin analogue might have different activities and potencies, but this is still to be completely determined [33]. A group from Germany studied the effects of curcumin *in vitro* on atrophic C2C12 cells, showing that sub toxic doses of curcumin successfully counteracted muscle atrophy [34]. Two other groups, one studying the effects of curcumin on rats bearing the Yoshida AH-130 ascites hepatoma cells (which is known to cause cachexia) [35], and another in mice bearing MAC16 tumor cells [36] did not show weight loss attenuation by curcumin found in the previously described animal study [30].

In the current study, patients treated with curcumin had a median survival from baseline of 189 days (95%CI 142-246), which was 110 days shorter than untreated patients (p=0.065). There are very few published clinical studies of curcumin in humans, and survival data is scarce. Epelbaum et al. [37] studied the effects of curcumin combined with gemcitabine for the treatment of advanced pancreatic cancer in 17 patients and reported a comparable median overall survival of 5 months (range 1-24 months) in the 11 patients who were considered evaluable [37]. Another group conducted a similar phase I/II study with the same drug combination in 21 patients with disease progression after gemcitabine treatment and with no prospect of further effective treatment and they found a similar median overall survival of 161 days (approximately 5 months) (95% confidence interval 109-223 days, approximately 4-7 months) [38].

In a retrospective analysis of 83 consecutive patients with pancreatic cancer referred to our Phase I program, it was shown that they had a median overall survival from referral of approximately 152 days (95%CI 99-186 days) [39]. This period is shorter than the results reported here for both study groups from the baseline image [189 days (95%CI 142-236 days) for patients treated with curcumin and 299 days (95%CI 240-357 days) for the controls]. For the patients in the treatment group, the dates of referral to the Phase I program and baseline image are very similar, so it is fair to state that patients who received curcumin had an overall survival around one month longer than the historic average of referred patients with the same diagnosis [39]. Of note, patients in the control group also had longer overall survival (five months more) than what was reported for patients with the same diagnosis seen at our Phase I program [39]. This is of interest because it might suggest an unforeseen selection bias caused by a systematic difference between patients referred to phase I and those who were not. It might be that, regardless of the matching efforts, patients in the control group had better health conditions at the inception point (time of first image), not being perceived by their physicians as candidates for the curcumin clinical trial and therefore not referred. It is conceivable that such bias might contribute to the difference in overall survival between patients in the treatment and control groups. In addition, it is important to mention that overall survival may be confounded by all treatments undertaken after the inception point, and that more than 50% of the patients in the control group were receiving oncologic treatment at the time of study entry and some of the patients in the treatment group received further treatments after being taken off the curcumin trial. Therefore, it is not possible to ascribe differences in survival only to the use of curcumin based solely on the data reported here. This is inherent to the retrospective design of the current study, despite all matching efforts.

In Figure 4, we demonstrate an apparent correlation between percent loss of total lean body mass and total adipose body mass with shorter survival in patients treated with curcumin, but this was not significant (p=0.202 and 0.09, respectively). Patients who were closer to death and receiving curcumin had greater loss of both total lean and adipose body masses, while patients in the control group presented an almost flat regression line, denoting that total lean and adipose body mass losses in these patients remained stable, regardless of the proximity to death. It seems as if patients who receive curcumin undergo a metabolic shift towards a more intense weight loss pattern when they are towards the end of their lives, but this shift does not reach statistical significance perhaps because of the small number of patients studied.

This study is not without limitations. The small sample size and its retrospective nature impair the ability of drawing definite conclusions. Additionally and also related to its retrospective methodology, this study is subject to selection bias, even though several measures were taken to minimize this risk (1:2 matching by several characteristics, random selection of controls).

In summary, this study demonstrated that both curcumin-treated and untreated patients lost weight due to a combination of fat and muscle depletion. Curcumin treated patients had greater losses in all body composition variables. Fat loss was the prominent feature in both groups, with different adipose compartments behaving differently: statistically significant fat loss occurred only in the subcutaneous area. Lean body mass loss also occurred and was significantly greater in the curcumin-treated patients. The use of complementary products is prevalent in patients with advanced malignancies [40]. A previous clinical trial in pancreatic cancer showed that two of 21 evaluable patients may have had salutary effects after curcumin treatment, including a patient with more than 18 months of stable disease and another patient with 73% tumor regression [32]. Recent studies suggest that pancreatic cancer is remarkably heterogeneous at the genomic level [41] and it may be that a small biologically definable subset of patients could be sensitive to curcumin. Even so, our current observations indicate that curcumin does not attenuate the development of sarcopenia in patients with advanced pancreatic cancer.

## MATERIALS AND METHODS

### Patient population

Patients treated with curcumin (“treatment” group) were obtained from a Phase II clinical trial conducted by our group to evaluate the safety and efficacy of a daily eight grams oral dose of the drug in the treatment of patients with advanced pancreatic cancer [32]. A total of 49 patients were treated on protocol between December/2004 and February/2006, and a subset of 22 patients was analyzed in the current study. Reasons for exclusion are depicted in Figure 1. The eligibility criteria for the original clinical trial included: (a) pathologically confirmed adenocarcinoma of the pancreas that is not amenable to curative surgical resection (includes locally advanced, metastatic, or recurrent disease), (b) Karnofsky Performance Status (KPS) ≥ 60 at study entry, (c) age ≥ 18 years, (d) adequate hematologic function as defined by an absolute neutrophil count ≥ 1,500/mm^2^ and platelet count ≥ 100,000/mm^3^, (e) adequate hepatic function as defined by a total bilirubin ≤ 2 times the upper limit of normality (ULN), alkaline phosphatase, ALT and/or AST ≤ 5 X ULN, (f) creatinine ≤ 2.0 mg/dL, (f) absence of brain metastases and (g) not have received radiation treatment within the 4 weeks before initiation of the clinical trial treatment. Treatment of patients on protocol as well as this analyses were performed according with The University of Texas M.D. Anderson Cancer Center Internal Review Board guidelines.

To be included in the current study, subjects in the treatment group also had to have one abdominal computed tomography (CT) image including the L3 vertebra level that was 28±7 days before the first day of treatment (baseline image) and one similar image within 60±20 days after the first day of treatment (follow up image).

A matched control group was obtained by searching the institutional databases for patients with advanced pancreatic cancer within the same calendar period which did not receive curcumin but would otherwise be eligible to participate in the clinical trial (according to the cited eligibility criteria) and that had two abdominal CT images including the L3 vertebra level separated by a range of 60±20 days. Patients in the control group were matched to patients in the treatment group according to gender, age, body mass index, time from advanced cancer diagnosis to baseline image, and number of prior therapies. To reduce the effect of any eventual selection bias, two controls were randomly selected per patient from the pool of potentially eligible patients (refer to Figure 1 for details about the control selection procedure).

### Data collection

Basic demographic data, date of advanced pancreatic cancer diagnosis (defined as locally advanced, recurrent, or metastatic), anticancer treatment history, medication use during study period, laboratory results, presence of ascites and/or edema, height, and weights were obtained by chart review. Eligible abdominal CT images were identified by chart review and downloaded.

### Body composition analysis

Body mass indices were calculated as usual by dividing the weight (in kilograms) by the height (in meters) squared [42]. Abdominal CT images at the level of the 3rd lumbar vertebra were used for body composition analysis. The use of this landmark has been previously described and validated against dual x-ray absorptiometry and bioimpedance analysis in healthy populations and in patients with advanced cancer [43–45]. Muscles, subcutaneous fat, and visceral fat were identified by a single assessor trained in the specific anatomy of these tissues, demarcated using previously described Hounsfield unit thresholds [46–48] and quantified with SliceOMatic software, version 4.3 (Tomovision, Montreal, QC, Canada). Total lean and fat body masses (LBM and FM, respectively) were estimated by applying the values obtained for muscularity and adiposity at L3 level to the Mourtzakis et al formulas (*LBM*(*kg*) = 0.30 × *skeletal muscle at L*3 (*cm*^2^) + 6.06 and *FM*(*kg*) = 0.042 × *fat tissue at L*3 (*cm*^2^) + 11.2) with demonstrated reliability (r=0.94, p<0.0001 and r=0.88, p<0.0001, respectively) [43]. A normalized skeletal muscle index was also calculated by dividing the area of muscle at L3 by the height squared. Patients were considered to be sarcopenic if they had a lumbar skeletal muscle index (skeletal muscle area at L3 divided by the height squared) lower than 38.5 cm^2^/m^2^ for women and lower than 52.4 cm^2^/m^2^ for men, as previously described [49].

### Statistical analyses

Descriptive statistics were used to summarize the data. Categorical variables were summarized by frequency. Differences in categorical variables were tested for statistical significance by using the chi-squared or Fisher exact tests, where appropriate. Differences in paired continuous variables were tested by paired t-tests when the underlying distribution was normal and by the Wilcoxon rank-sum test when normality could not be assumed. Statistical significance for differences between independent continuous variables was evaluated by t-tests and Mann-Whitney tests depending if normality was respectively assumed or not. Survival analyses were conducted using Kaplan Meier plots with log-rank analyses. Patients for whom date of death was not found were censored at the time of last follow up. Differences were deemed to be statistically significant when the p values were less than or equal to 0.05. Analyses were performed using SPSS v. 16 (SPSS Inc, Chicago, IL).

## References

[1] Radbruch L, Elsner F, Trottenberg P, Strasser F, Fearon K (2010). Clinical practice guidelines on cancer cachexia in advanced cancer patients.

[2] Fearon K, Strasser F, Anker SD, Bosaeus I, Bruera E, Fainsinger RL, Jatoi A, Loprinzi C, MacDonald N, Mantovani G, Davis M, Muscaritoli M, Ottery F, Radbruch L, Ravasco P, Walsh D (2011). Definition and classification of cancer cachexia: an international consensus. Lancet Oncol.

[3] Dewys WD, Begg C, Lavin PT, Band PR, Bennett JM, Bertino JR, Cohen MH, Douglass HO, Engstrom PF, Ezdinli EZ, Horton J, Johnson GJ, Moertel CG, Oken MM, Perlia C, Rosenbaum C (1980). Prognostic effect of weight loss prior to chemotherapy in cancer patients. Eastern Cooperative Oncology Group. Am J Med.

[4] Wallengren O, Iresjo BM, Lundholm K, Bosaeus I (2015). Loss of muscle mass in the end of life in patients with advanced cancer. Support Care Cancer.

[5] Skipworth RJ, Stewart GD, Dejong CH, Preston T, Fearon KC (2007). Pathophysiology of cancer cachexia: much more than host-tumour interaction?. Clin Nutr.

[6] Deans DA, Wigmore SJ, de Beaux AC, Paterson-Brown S, Garden OJ, Fearon KC (2007). Clinical prognostic scoring system to aid decision-making in gastro-oesophageal cancer. Br J Surg.

[7] Fearon KC, Voss AC, Hustead DS (2006). Definition of cancer cachexia: effect of weight loss, reduced food intake, and systemic inflammation on functional status and prognosis. Am J Clin Nutr.

[8] Dahele M, Skipworth RJ, Wall L, Voss A, Preston T, Fearon KC (2007). Objective physical activity and self-reported quality of life in patients receiving palliative chemotherapy. J Pain Symptom Manage.

[9] LeBlanc TW, Nipp RD, Rushing CN, Samsa GP, Locke SC, Kamal AH, Cella DF, Abernethy AP (2015). Correlation between the international consensus definition of the Cancer Anorexia-Cachexia Syndrome (CACS) and patient-centered outcomes in advanced non-small cell lung cancer. J Pain Symptom Manage.

[10] Wesseltoft-Rao N, Hjermstad MJ, Ikdahl T, Dajani O, Ulven SM, Iversen PO, Bye A (2015). Comparing two classifications of cancer cachexia and their association with survival in patients with unresected pancreatic cancer. Nutrition and cancer.

[11] Deans C, Wigmore SJ (2005). Systemic inflammation, cachexia and prognosis in patients with cancer. Curr Opin Clin Nutr Metab Care.

[12] Singh S (2007). From exotic spice to modern drug?. Cell.

[13] Araujo CC, Leon LL (2001). Biological activities of Curcuma longa L. Mem Inst Oswaldo Cruz.

[14] Fu S, Kurzrock R (2010). Development of curcumin as an epigenetic agent. Cancer.

[15] Lestari ML, Indrayanto G (2014). Curcumin. Profiles of drug substances, excipients, and related methodology.

[16] Kunnumakkara AB, Anand P, Aggarwal BB (2008). Curcumin inhibits proliferation, invasion, angiogenesis and metastasis of different cancers through interaction with multiple cell signaling proteins. Cancer Lett.

[17] Li L, Aggarwal BB, Shishodia S, Abbruzzese J, Kurzrock R (2004). Nuclear factor-kappaB and IkappaB kinase are constitutively active in human pancreatic cells, and their down-regulation by curcumin (diferuloylmethane) is associated with the suppression of proliferation and the induction of apoptosis. Cancer.

[18] Sun M, Estrov Z, Ji Y, Coombes KR, Harris DH, Kurzrock R (2008). Curcumin (diferuloylmethane) alters the expression profiles of microRNAs in human pancreatic cancer cells. Mol Cancer Ther.

[19] Rahmani AH, Al Zohairy MA, Aly SM, Khan MA (2014). Curcumin: a potential candidate in prevention of cancer via modulation of molecular pathways. BioMed research international.

[20] Aggarwal BB, Sung B (2009). Pharmacological basis for the role of curcumin in chronic diseases: an age-old spice with modern targets. Trends Pharmacol Sci.

[21] Aggarwal BB, Van Kuiken ME, Iyer LH, Harikumar KB, Sung B (2009). Molecular targets of nutraceuticals derived from dietary spices: potential role in suppression of inflammation and tumorigenesis. Exp Biol Med (Maywood).

[22] Shishodia S, Singh T, Chaturvedi MM (2007). Modulation of transcription factors by curcumin. Adv Exp Med Biol.

[23] Wigmore SJ, Plester CE, Richardson RA, Fearon KC (1997). Changes in nutritional status associated with unresectable pancreatic cancer. Br J Cancer.

[24] Tan BH, Birdsell LA, Martin L, Baracos VE, Fearon KC (2009). Sarcopenia in an overweight or obese patient is an adverse prognostic factor in pancreatic cancer. Clin Cancer Res.

[25] Parsons HA, Baracos V, Dhillon N, Kurzrock R (2010). A Preliminary Investigation of Body Composition, Symptom Burden and Survival in a Phase I Clinical Trials Service (abstr). Support Care Cancer.

[26] Veasey Rodrigues H, Baracos VE, Wheler JJ, Parsons HA, Hong DS, Naing A, Fu S, Falchoock G, Tsimberidou AM, Piha-Paul S, Chisholm G, Kurzrock R (2013). Body composition and survival in the early clinical trials setting. European journal of cancer (Oxford, England : 1990).

[27] Joglekar S, Asghar A, Mott SL, Johnson BE, Button AM, Clark E, Mezhir JJ (2015). Sarcopenia is an independent predictor of complications following pancreatectomy for adenocarcinoma. Journal of surgical oncology.

[28] Shehzad A, Khan S, Sup Lee Y (2012). Curcumin molecular targets in obesity and obesity-related cancers. Future Oncol.

[29] Shehzad A, Ha T, Subhan F, Lee YS (2011). New mechanisms and the anti-inflammatory role of curcumin in obesity and obesity-related metabolic diseases. European journal of nutrition.

[30] Siddiqui RA, Hassan S, Harvey KA, Rasool T, Das T, Mukerji P, DeMichele S (2009). Attenuation of proteolysis and muscle wasting by curcumin c3 complex in MAC16 colon tumour-bearing mice. Br J Nutr.

[31] van der Worp HB, Howells DW, Sena ES, Porritt MJ, Rewell S, O'Collins V, Macleod MR (2010). Can animal models of disease reliably inform human studies?. PLoS Med.

[32] Dhillon N, Aggarwal BB, Newman RA, Wolff RA, Kunnumakkara AB, Abbruzzese JL, Ng CS, Badmaev V, Kurzrock R (2008). Phase II trial of curcumin in patients with advanced pancreatic cancer. Clin Cancer Res.

[33] Anand P, Thomas SG, Kunnumakkara AB, Sundaram C, Harikumar KB, Sung B, Tharakan ST, Misra K, Priyadarsini IK, Rajasekharan KN, Aggarwal BB (2008). Biological activities of curcumin and its analogues (Congeners) made by man and Mother Nature. Biochem Pharmacol.

[34] Oelkrug C, Lange CM, Wenzel E, Fricke S, Hartke M, Simasi J, Schubert A (2014). Analysis of the tumoricidal and anti-cachectic potential of curcumin. Anticancer research.

[35] Busquets S, Carbo N, Almendro V, Quiles MT, Lopez-Soriano FJ, Argiles JM (2001). Curcumin, a natural product present in turmeric, decreases tumor growth but does not behave as an anticachectic compound in a rat model. Cancer Lett.

[36] Wyke SM, Russell ST, Tisdale MJ (2004). Induction of proteasome expression in skeletal muscle is attenuated by inhibitors of NF-kappaB activation. Br J Cancer.

[37] Epelbaum R, Schaffer M, Vizel B, Badmaev V, Bar-Sela G (2010). Curcumin and gemcitabine in patients with advanced pancreatic cancer. Nutr Cancer.

[38] Kanai M, Yoshimura K, Asada M, Imaizumi A, Suzuki C, Matsumoto S, Nishimura T, Mori Y, Masui T, Kawaguchi Y, Yanagihara K, Yazumi S, Chiba T, Guha S, Aggarwal BB (2011). A phase I/II study of gemcitabine-based chemotherapy plus curcumin for patients with gemcitabine-resistant pancreatic cancer. Cancer chemotherapy and pharmacology.

[39] Vaklavas C, Tsimberidou AM, Wen S, Hong D, Wheler J, Ng CS, Naing A, Uehara C, Wolff RA, Kurzrock R (2011). Phase 1 clinical trials in 83 patients with pancreatic cancer: The M. D. Anderson Cancer Center experience. Cancer.

[40] Naing A, Stephen SK, Frenkel M, Chandhasin C, Hong DS, Lei X, Falchook G, Wheler JJ, Fu S, Kurzrock R (2011). Prevalence of complementary medicine use in a phase 1 clinical trials program: the MD Anderson Cancer Center Experience. Cancer.

[41] Heestand GM, Kurzrock R (2015). Molecular landscape of pancreatic cancer: implications for current clinical trials. Oncotarget.

[42] Billewicz WZ, Kemsley WF, Thomson AM (1962). Indices of adiposity. Br J Prev Soc Med.

[43] Mourtzakis M, Prado CM, Lieffers JR, Reiman T, McCargar LJ, Baracos VE (2008). A practical and precise approach to quantification of body composition in cancer patients using computed tomography images acquired during routine care. Appl Physiol Nutr Metab.

[44] Shen W, Punyanitya M, Wang Z, Gallagher D, St-Onge MP, Albu J, Heymsfield SB, Heshka S (2004). Total body skeletal muscle and adipose tissue volumes: estimation from a single abdominal cross-sectional image. J Appl Physiol.

[45] Shen W, Punyanitya M, Wang Z, Gallagher D, St-Onge MP, Albu J, Heymsfield SB, Heshka S (2004). Visceral adipose tissue: relations between single-slice areas and total volume. Am J Clin Nutr.

[46] Miller KD, Jones E, Yanovski JA, Shankar R, Feuerstein I, Falloon J (1998). Visceral abdominal-fat accumulation associated with use of indinavir. Lancet.

[47] Heymsfield SB, Smith R, Aulet M, Bensen B, Lichtman S, Wang J, Pierson RN (1990). Appendicular skeletal muscle mass: measurement by dual-photon absorptiometry. Am J Clin Nutr.

[48] Mitsiopoulos N, Baumgartner RN, Heymsfield SB, Lyons W, Gallagher D, Ross R (1998). Cadaver validation of skeletal muscle measurement by magnetic resonance imaging and computerized tomography. J Appl Physiol.

[49] Prado CM, Lieffers JR, McCargar LJ, Reiman T, Sawyer MB, Martin L, Baracos VE (2008). Prevalence and clinical implications of sarcopenic obesity in patients with solid tumours of the respiratory and gastrointestinal tracts: a population-based study. Lancet Oncol.

